# The Role of Medical Structural Genomics in Discovering New Drugs for Infectious Diseases

**DOI:** 10.1371/journal.pcbi.1000530

**Published:** 2009-10-26

**Authors:** Wesley C. Van Voorhis, Wim G. J. Hol, Peter J. Myler, Lance J. Stewart

**Affiliations:** 1 Department of Medicine, University of Washington, Seattle, Washington, United States of America; 2 Department of Biochemistry, University of Washington, Seattle, Washington, United States of America; 3 Seattle Biomedical Research Institute, Seattle, Washington, United States of America; 4 Department of Global Health, University of Washington, Seattle, Washington, United States of America; 5 Department of Medical Education and Biomedical Informatics, University of Washington, Seattle, Washington, United States of America; 6 deCODE biostructures, Bainbridge Island, Washington, United States of America; Massachusetts Institute of Technology, United States of America

## Introduction

Whether we think of Alzheimer's disease, microbial infection, or any other modern-day disease, new medicines are urgently needed. The number of new drugs registered since the advent of genomics, however, has not lived up to expectations. One recent review revealed that over 70 high-throughput biochemical screens against genetically validated drug targets in bacteria failed to yield a single candidate that could be tested in the clinic [1]. The reasons for the failure of high-throughput biochemical screens are not completely clear, but it could reflect the limited diversity of chemical libraries used and/or the absence of structural information for many of the targets. Indeed, structure-based drug design is playing a growing role in modern drug discovery, with numerous approved drugs tracing their origins, at least in part, to the use of structural information from X-ray crystallography or nuclear magnetic resonance (NMR) analysis of protein targets and their ligand-bound complexes. Although it is beyond the scope of this brief overview to present a comprehensive list of structures that have led to useful drugs, Table 1 lists some examples in which protein structure information has provided insights to the design and development of new therapeutic entities. These cases include both novel drug design based on native and ligand-bound structures and optimization of inhibitors based on the binding mode revealed by the structures of inhibitor–target complexes. These approaches have allowed increased affinity for the target and/or improvement of pharmacological properties while maintaining target affinity.

**Table 1:** Examples of how target protein structure can assist drug discovery and development.

Source	Target Protein	Approach	Reference(s)
**HIV**	gp41	Structure led to strategies that target viral entry.	[43]–[45]
**HIV**	Protease	Protease–inhibitor complexes allowed lead optimization.	[46]–[52]
**HIV**	Reverse transcriptase	Non-nucleoside inhibitor complexes led to drug design that targets pockets outside the enzyme's active site.	[53]–[55]
**Influenza virus**	Neuraminidase	Complex with a transition state analog led to inhalable and orally active neuraminidase inhibitors.	[56]–[59]
**Rhinovirus**	Coat protein	Small fatty acid molecules bound in hydrophobic pocket led to new strategies of antiviral drug design.	[60]
***Vibrio***	Cholera toxin	Five receptor-binding sites provided inspiration for design of novel multivalent inhibitors.	[61]
**Bacteria**	Peptide deformylase	Protein–inhibitor complexes led to macrocyclic compounds with improved potency, selectivity and metabolic stability.	[62]
***Trypanosoma***	GAPDH	Novel adenosine analogs showed enhanced selectivity towards the parasite target versus human protein.	[63],[64]
**Human**	Cyclophilin and calcineurin	A ternary complex with cyclosporine A led to insights into its immunosuppressive activity.	[65]
**Human**	Renin	The ligand-bound structure allowed design and improvement of orally active non-peptide inhibitors to regulate blood pressure.	[66]
**Human**	Coagulation factor Xa	Structure-based design led to improved pharmacological anticoagulant properties in a primate model.	[67]
**Human**	Adenosine deaminase	Optimization of a non-nucleoside inhibitor led to an orally active anti-inflammatory compound in a rat model.	[68]
**Human**	Kinases	Structures of kinases provided a basis to improve and design new therapeutics for various human diseases including cancer.	[69]

With the increasing availability of complete human and pathogen genome sequences and the substantial progress in structure determination methods, it is no surprise that the field of “structural genomics” has emerged recently. Its aim is to solve as many useful protein structures as possible from the entire genome of a single organism or group of related organisms. Over the past ten years, over 20 structural genomics initiatives have begun around the world (Table 2). The impact of these efforts on structural biology has been substantial, both in the sheer number of new structures and, perhaps even more importantly, in the development of new methodologies, especially the use of robotics and informatics to generate and capture data in a systematic way [2]. Over the next five years, thousands of new protein structures, many bound to their ligands, will be elucidated; laying the groundwork for structure-based design and development of new and improved chemotherapeutic agents against pathogen proteins. Here, we will focus on the intersection of structural biology with chemistry and biology—a field called “medical structural genomics”—particularly on how the structures of medically relevant drug targets in pathogens can serve as a starting point for inhibitor design and drug development. We argue that the pharmaceutical industry should be persuaded to complement the publicly funded structural genomics initiatives by making public the structural coordinates of their drug targets for important infectious disease organisms in a timely fashion and by developing public–private partnerships to provide the maximal synergy between target validation, structure determination, and hit-to-lead development.

**Table 2:** Structural genomics projects worldwide submitting to the Protein Data Bank.

Name	URL	Target Focus
Berkeley Structural Genomics Center (BSGC)	http://www.strgen.org/	Near complete coverage of *Mycoplasma* genome
Center for Eukaryotic Structural Genomics (CESG)	http://www.uwstructuralgenomics.org/	PSI Center—Eukaryotic bottlenecks, specifically solubility
Center for Structural Genomics of Infectious Disease (CSGID)	http://csgid.org/csgid/	Medically relevant infectious disease targets
Center for Structure of Membrane Proteins (CSMP)	http://csmp.ucsf.edu/index.htm	PSI Center—Bacterial and human membrane proteins
Integrated Center for Structure and Function Innovation (ISFI)	htp://techcenter.mbi.ucla.edu/	PSI Center—Protein solubility and crystallization improvement
Israel Structural Proteomics Center	http://www.weizmann.ac.il/ISPC/	Member of Structural Proteomics in Europe (see below)
Joint Center for Structural Genomics (JCSG)	http://www.jcsg.org/	PSI Center—High-throughput pipeline development and operation
Marseilles Structural Genomics Program	http://www.afmb.univ-mrs.fr/rubrique93.html	Human health
Medical Structural Genomics of Pathogenic Protozoa (MSGPP)	http://www.msgpp.org/	Structural and functional genomics of ten species of pathogenic protozoa
Montreal-Kingston Bacterial Structural Genomics Initiative (BSGI)	http://euler.bri.nrc.ca/brimsg/bsgi.html	ORFs from pathogenic and nonpathogenic bacterial strains
Mycobacterium Tuberculosis Structural Genomics Consortium (TBsgc)	http://www.doe-mbi.ucla.edu/TB/	*Mycobacterium tuberculosis*—To understand pathogenesis and for structure-based drug design
Mycobacterium Tuberculosis Structural Proteomics Project (X-MTB)	http://webclu.bio.wzw.tum.de/binfo/proj/mtb/	35 *Mycobacterium tuberculosis* targets to identify five for drug development
New York SGX Research Center for Structural Genomics (NYSGXRC)	http://www.nysgrc.org/nysgrc/	PSI Center—High-throughput pipeline development and operation
Ontario Center for Structural Proteomics (OCSP)	http://www.uhnres.utoronto.ca/centres/proteomics/	Enzymatic activity characterization
Oxford Protein Production Facility	http://www.oppf.ox.ac.uk/OPPF/	Human and pathogen targets of biomedical relevance
RIKEN Structural Genomics/Proteomics Initiative	http://www.rsgi.riken.jp/rsgi_e/	Protein functional networks
Seattle Structural Genomics Center for Infectious Disease (SSGCID)	http://www.ssgcid.org/	Medically relevant infectious disease targets
Southeast Collaboratory for Structural Genomics	http://www.secsg.org/	High-throughput eukaryotic genome-scan methods development
Structural Genomics of Pathogenic Protozoa	http://www.sgpp.org/	PSI Center - Three-dimensional structures of proteins from four major pathogenic protozoa
Structural Proteomics in Europe (SPINE)	http://www.spineurope.org/	Structures of medically relevant proteins and protein complexes
Structural Proteomics in Europe 2-Complexes (SPINE2 - Complexes)	http://www.spine2.eu/SPINE2/	Structures of protein complexes from medically relevant signaling pathways
Structural Genomics Consortium	http://www.thesgc.org/	Medically relevant human and pathogen proteins
Structure 2 Function Project	http://s2f.umbi.umd.edu/	Poorly characterized and hypothetical protein targets
The Accelerated Technologies Center for Gene to 3D Structure	http://atcg3d.org/default.aspx	PSI Center—Technologies development of X-ray source, synthetic gene design, and microfluidic crystallization
The Midwest Center for Structural Genomics (MCSG)	http://www.mcsg.anl.gov/	PSI Center—High-throughput methods development and operation
The Northeast Structural Genomics Consortium (NESG)	http://www.nesg.org/	PSI Center—Protein domains, network families, biomedical relevance

Note: Some centers with fewer than ten released structures in the PDB (www.rcsb.org/pdb/) are not shown.

PSI, Protein Structure Initiative.

## Target Selection

A prerequisite of medical structural genomics is that the proteins whose structures are determined must be well-validated as good drug targets. The term “drugability” is often used to loosely describe how tractable any given target is for the development of a drug candidate. For infectious organisms, one key factor in defining drugability is that the target protein be essential for survival of the microbe. While essentiality has traditionally been defined using techniques such as “gene knockout” and RNA interference, these are not always feasible and should be complemented by chemical biology approaches (see below). Furthermore, the meaningfulness of these experiments can often be difficult to assess, since the interplay of host and pathogen is complex and full of surprises. For example, tremendous effort has been devoted recently to the development of antagonists for targets in the fatty acid biosynthesis pathway of bacteria [3]. Potent drug-like molecules with high bioavailability have been developed that can effectively shut down bacterial replication in vitro. These compounds were found to be ineffective in subsequent animal testing, however, because fatty acids are quite abundant in vertebrates, so bacteria can secure these host molecules for their survival and growth even if their own fatty acid biosynthesis pathways are blocked [4]. Thus, to improve target selection for medical structural genomics, it will be important to collaborate with chemical biology groups to undertake screening campaigns to identify compounds that cause the death of a pathogen under the appropriate assay conditions [5].

If the target protein of a drug is known, medical structural genomics offers a rapid and efficient way to obtain ligand-bound structures by using high-throughput X-ray crystallography and/or NMR. Conversely, when the target of a cell-active compound is unknown, medical structural genomics efforts provide purified protein for many potential drug targets that can be screened for interaction with the active compound by a number of biophysical methods (such as thermal stability [6]). The Medicinal Structural Genomics of Protozoan Pathogens (MSGPP, http://www.msgpp.org/) initiative has already begun such an effort by screening thousands of anti-malaria compounds against 67 potential *Plasmodium falciparum* targets expressed in bacteria (WC Van Voorhis, unpublished data). These approaches aim to generate knowledge about the biological effect of a small molecule on a target protein. Follow-up experiments are then needed to test the activity of this compound in live organisms in order to validate the target; this valuable “chemical validation” makes the target much more likely to be drugable, and thus worthy of more intensive effort. The future will likely see more medical structural genomics centers working with chemical biology groups that have collections of “phenotype-defined” compounds (i.e., those with known anti-pathogen activity). The result will be synergistic target validation and hit-to-lead development using structure-based drug design.

## Fragment-Based Drug Discovery

**Figure 1:**
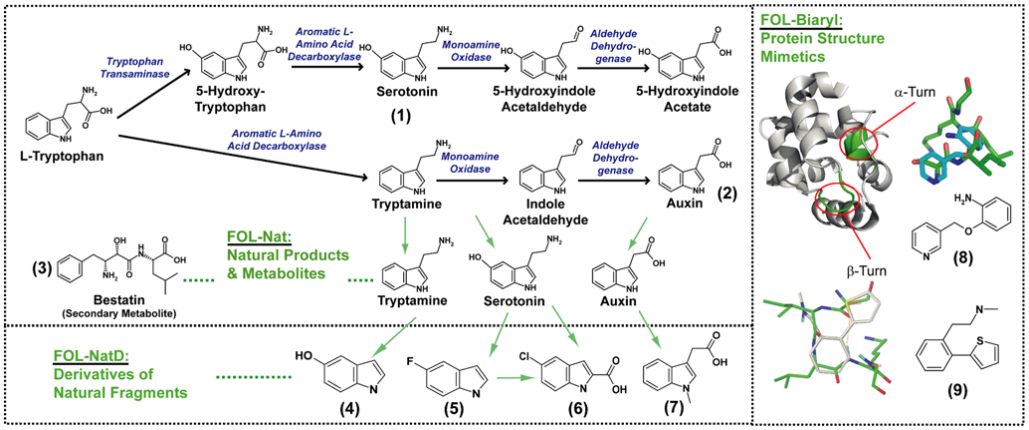
Conceptual organization of the deCODE biostructures Fragments of Life library. The current ∼1,400-compound library contains chemically tractable natural small molecule metabolites (FOL-Nat), metabolite-like compounds and their bioisosteres (FOL-NatD), and biaryl mimetics of protein architecture (FOL-Biaryl). The FOL-Nat members include any natural molecule of molecular weight <350 daltons that exists as a substrate, natural product, or allosteric regulator of any metabolic pathway in any cell type, such as the biosynthetic pathways for the neurotransmitter serotonin (**1**) and the plant hormone auxin (**2**). The FOL-Nat members also include secondary metabolites such as bestatin (**3**), a secondary metabolite of *Streptomyces olivoreticuli* [38]. FOL-NatD fragments are defined as heteroatom-containing derivatives, isosteres, or analogs of any FOL-Nat molecule. For example, fragments **4–7** contain the indole scaffold, which is known to be a privileged building block for drug molecules [39]. To emulate protein architecture, the FOL-Biaryl fragments were selected from a variety of biaryl compounds that are potential mimics of protein α, β, or γ turns [40]–[42]. These include a compound (**8**) whose structure in an energy-minimized state can be seen to mimic the architecture on an α-turn of a protein structure (here, residues Ser65-Ile66-Leu67-Lys68 of PDB ID:1RTP) and, similarly, a compound (**9**) whose structure mimics the β-turn of a protein structure (residues Ala20-Ala21-Asp22-Ser23).

Fragment-based drug discovery has rapidly gained interest within the pharmaceutical industry (reviewed in [7] with roots of 128-compound cocktails in [8]), as an alternative to expensive and sometimes inefficient high-throughput screening methods for hit identification and optimization [9]. The general concept of fragment-based drug discovery involves screening libraries of “rule-of-three” compounds [10] against target macromolecules by using a variety of methods including X-ray crystallography, NMR, surface plasmon resonance, differential thermal denaturation, fluorescence polarization, and other techniques [7], [11]–[14]. The rule of three consists of molecular weight <300 daltons, ≤3 rotatable bonds, ≤3 hydrogen bond donors/acceptors, and Clog P (calculated log of octanol/water partition coefficient) <3. These compounds generally include fragments or “building blocks” of available drugs, on the assumption that these fragments are more likely to be “drug-like.” Fragment-based drug discovery has been used by commercial and academic groups, including our own, and has led to a number of leads for further drug development [15]. At deCODE biostructures, a partner in the Seattle Structural Genomics Center for Infectious Disease (SSGCID, http://www.ssgcid.org/) consortium, the approach to assembling a fragment library has been somewhat different. The Fragments of Life (FOL) library (Figure 1) is a collection of approximately 1,400 structurally diverse small molecules found in the cellular environment, metabolites, natural products, and their derivatives or isosteres (molecules of similar size containing the same number and types of atoms). Also included in the FOL library are a series of biaryl small molecules (which contain two tethered five- or six-membered ring structures) that mimic protein secondary structure elements (e.g., α-helical turns). Thus, this fragment set is useful for targeting both the active sites of enzymes and more complex protein surfaces including allosteric small molecule binding sites and protein–protein interfaces [16].

## Targeting Oligomeric Enzymes

**Figure 2:**
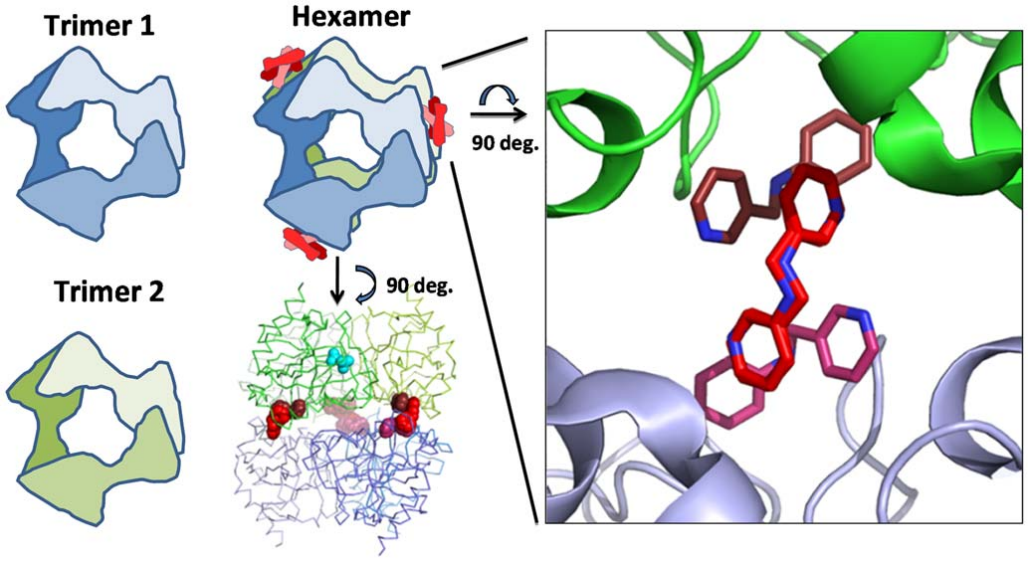
*B. pseudomallei* inorganic pyrophosphatase with bound ligand at an oligomeric interface. Homo-hexameric bacterial inorganic pyrophosphatase is a dimer of trimers (blue and green). The illustration shows the hexamer structure in a complex with three ligand fragment molecules (red spheres and stick structures represent fragment FOL 110), each of which is located at one of three “dimer of trimer” interfaces (1.5 ligands per monomer) (PDBID:3EJ0). The location of one pyrophosphate substrate (cyan spheres) at the active site of one of the monomers is indicated here based on the superimposed structure of the hexamer with pyrophosphate bound in the active site (PDBID:3EIY). The binding sites of the ligands (red) are clearly seen in a pocket formed by the homo-oligomeric assemblage, which is distant from the active site where pyrophosphate (cyan) binds.

Protein–protein interaction and assemblies, ranging from simple dimers to extremely complex arrangements as seen in the ribosome or the nuclear pore complex, form the basis of most biological processes, and there are usually numerous points of contact between the macromolecules involved. Yet the protein–protein interfaces formed by oligomerization are not necessarily accompanied by a large gain in free energy, and small molecules have been shown to prevent critical protein–protein interactions [17]. These findings have prompted recent discussion of a structure-based approach aimed at developing novel small-molecule antibiotics that modulate protein activity by binding to an interface between subunits within multi-protein complexes [18]. The bacterial enzyme inorganic pyrophosphatase may serve as an example for this approach, since it exists in a hexameric state that requires conformational flexibility for its essential role in converting inorganic pyrophosphate into phosphate [19]–[21]. Moreover, whereas all bacterial inorganic pyrophosphatases function as a homohexamer, the eukaryotic cytosolic and mitochondrial inorganic pyrophosphatases function as homodimers [21]. Hence eukaryotic inorganic pyrophosphatases have different oligomeric interfaces than those of bacterial enzymes. This suggests that it may be possible to inhibit the bacterial inorganic pyrophosphatase safely by targeting its oligomeric state rather than its highly conserved active site. A similar approach has recently been used to identify species-specific modulators of porphobilinogen synthase (PBGS) activity [22]. SSGCID has solved the high-resolution X-ray crystal structure of inorganic pyrophosphatase from the pathogenic bacterium *Burkholderia pseudomallei*, and a subsequent FOL screen of this target identified several fragments that specifically bind at multiple oligomerization pockets in a molecular interface between the two trimers of the homohexamer (Figure 2). While these fragments remain to be validated in terms of their species-specific inhibition of inorganic pyrophosphatase activity, they represent potential starting points for the development of novel antibiotics.

## Industry-Generated Structures and the Protein Data Bank

As we have seen above, protein structure information is the bread and butter of structure-based drug discovery. Structural genomics projects (Table 2) have substantially increased the number of protein structures solved and have made this information freely and openly available (i.e., at no cost and without restriction by copyright or other constraints) by depositing it in the Protein Data Bank (PDB) [23]. Most publishers have policies that require authors to deposit structural data in the PDB at the time of publication, so structures determined by academic researchers worldwide are, for the most part, well disseminated. By contrast, the pharmaceutical industry is sitting on a mountain of structural data for protein–ligand complexes from globally important pathogens, which is not available to the wider scientific community. The secrecy engendered by the current economic incentives driving drug discovery in the commercial sector has led to a substantial waste of precious resources through duplication of effort and inability to learn from others' successes and failures. The situation is unlikely to change without a concerted effort to find ways to overcome the financial and intellectual property barriers that prevent dissemination of this information. A recent publication suggested that open access industry–academia partnerships may provide one possible model [24]. We propose that the United States National Institutes of Health, along with other national and international research-funding agencies, issue calls for proposals that will fund the transfer of the highly valuable structural information from corporate databases into the PDB. Such an effort would obviously require discussion with industrial parties to negotiate mutually acceptable policies and mechanisms for the deposition of these structures in the public databases. These might include relaxation of release standards for industrial entities, such that structural information could be safely deposited in PDB at the time of structure determination and released only at a later date more appropriate for protection of intellectual property.

## Challenges for the Future

We are currently witnessing an explosion in technological and computational advances in structural genomics, with protein structures of hundreds or thousands of medically relevant targets from infectious disease organisms likely to be available over the next few years. This new information provides both academic and for-profit scientists with an unprecedented opportunity to accelerate the development of new and improved chemotherapeutic agents against these pathogens. One major challenge will be the adaptation of existing fragment-based drug design methods to match the scale of the structural genomics era. New high-throughput methods need to be developed for fragment-screening to enhance the success rate for protein–ligand structure determination.

Major attention is also needed to the development of fully automated, very high throughput crystal growth screening methods to elucidate the binding of well-selected compounds to medically relevant targets. These screens need to cover many (up to 100) protein variants [25],[26], 1,000–10,000 different small molecule compounds, and approximately 1,000 different crystal growth conditions [27], resulting in 10^8^ to 10^9^ conditions to be tested for a single drug target. Obviously, this will require development of even smaller volume assays than those currently in use [28]–[31]—down to the low picoliters—and automated detection of crystals in the millions of crystallization chambers [32]–[34]. Further development of automated capillary crystallization methods [35] might provide another way to achieve the very high throughput crystal screening required for reaching the full power of medical structural genomics in the future. Cryoprotection of the crystals is a specific hurdle, although it might be possible to routinely collect and merge partial datasets from multiple crystals under non-cryo conditions. Alternatively, the use of micromeshes [36],[37] and further miniaturization of trays and other crystal screening tools may allow cryoprotection of many crystals simultaneously.

In addition, existing databases will need to be modified to allow easy dissemination of the results from these fragment screens, and a serious effort should be made to persuade small and big pharma to release coordinates of drug targets from globally important infectious disease organisms. It will also be critical (but challenging) for structural biologists to collaborate with medicinal chemists and molecular biologists to turn these fragment from promising leads to effective drugs. Together, these steps should begin to release a flood of structures that provide a tremendous resource for improving health in rich and poor countries alike.
